# Efficacy and Safety of *Gwakhyangjeonggi-San* Retention Enema in Normal Rats and Spontaneously Hypertensive Rats

**DOI:** 10.1155/2013/765914

**Published:** 2013-06-17

**Authors:** Eunyoung Song, Euiju Lee, Yongmin Bu, Junhee Lee, Seungwon Shin, Junghee Yoo, Jaewoo Park, Jinhyeok Kwon

**Affiliations:** ^1^Department of Sasang Consitutional Medicine, Graduate School of Clinical Korean Medicine, Kyung Hee University, Seoul 130-702, Republic of Korea; ^2^Department of Sasang Constitutional Medicine, College of Korean Medicine, Kyung Hee University, Seoul 130-702, Republic of Korea; ^3^Department of Herbal Pharmacology, College of Korean Medicine, Kyung Hee University, Seoul 130-702, Republic of Korea; ^4^Department of Spleen System, College of Korean Medicine, Kyung Hee University, Seoul 130-702, Republic of Korea

## Abstract

The purpose of this study is to establish a protocol of retention-enema experiments and evaluate the antihypertensive effect and the safety of *Gwakhyangjeonggi-san* retention enema. Normal and spontaneously hypertensive rats (SHRs) were divided into treatment and control groups, respectively. We applied the *Gwakhyangjeonggi-san* extract by decoction and 0.9% NaCl in each group, estimated the blood pressure and body weight, and performed HPLC analysis. ALT, AST, BUN, and creatinine were examined. The systolic blood pressure within each group in normal rats differed significantly in time effect, and so did the diastolic blood pressure in the treatment group of normal rats. The systolic, diastolic, and mean blood pressure showed significant differences in group effect in the treatment group of the SHRs. The time effect of the body weight in both groups of normal rats differed significantly, so did group × time and time effects in both groups of SHRs. AST, ALT, BUN, and creatinine showed no significant difference between groups. We concluded that the *Gwakhyangjeonggi-san* retention enema has a hypotensive effect in normal rats within the regular range of blood pressure, but an antihypertensive effect in SHRs. Also, the intervention is safe and does not affect the liver and kidney functions in normal rats.

## 1. Introduction

An enema is a procedure to infuse or drip medication into the rectum through the anus. Decoctions or liquids of medication are usually used as the external treatment. Especially, retention enema is the way to keep drugs (normal saline, herbal medicine, or coffee, etc.) in the rectum for a relatively long time [1]. 

Bloemen et al. showed that butyrate enemas enhance anastomotic strength of the intestine [2], de Sounza et al. investigated the budesonide and probiotics enemas which are effective to diminish colitis [3], and Bae et al. suggested the anal therapy using Syzygium aromaticum could regulate immediate-type hypersensitivity or anaphylaxis [4].

Only a few investigations involved in retention enema have been conducted. According to the preceding experimental researches, Sprague-Dawley rats [5], mice [6], and Wister rats [7, 8] were used in the experiments of retention enema. And pediatric foley catheters [5], stomach nodes [9], or nylon hoses [7] were used to insert test liquids. The retention time was 15 seconds [7], 30 seconds [3, 10], or 10 minutes [6]. The target disease of most researches was colitis [3, 5, 7, 10]. These show that the method of the retention enema experiments is not unified, nor systematic. 

Retention enema is applied to a broader range of diseases including colon-related disorders, cancers, hypertension, and prehypertension in Asian counties. Recently, retention enema was reported as an effective treatment to prevent prehypertension patients from hypertension [11]. However, it is necessary to find out the efficacy and the safety of retention enema adopted in patients with prehypertension or hypertension.

Korea National Health & Nutrition Examination Survey announced that, among the Korean adults over 30 years, 21.8% of prehypertensive patients and 28.9% of hypertensive patients were reported in 2010, which means that a half of the people over age 30 had hypertension or the risk of developing hypertension [12]. According to another research, 16.6% of individuals of normal blood pressure progressed to hypertension in 5 years, whereas 32.7% of those of prehypertension did so [13]. A cohort research including 169, 871 Chinese people over 40 years old revealed that the risk to develop cardiovascular disorders is higher in the prehypertension group than in normal blood pressure group (relative risk = 1.34), and it is effective to treat prehypertension in patients with diabetes or cardiovascular disorders [14]. This means that it is necessary to detect and regulate the prehypertension early because it progresses easily to hypertension and elevates the occurrence risk of cardiovascular disorders.

In Kim's manual that investigated the clinical effectiveness of retention enema in the hypertension and prehypertension groups, *Gwakhyangjeonggi-san* was used as a test liquid. *Gwakhyangjeonggi-san* is known to block the Ca^2+^ inflow, which results in the vasorelaxant effect by NO action isolated from blood vessels [15]. Expecting that *Gwakhyangjeonggi-san* could act as a vasorelaxant effector in vasoconstrictive diseases such as hypertension [15], we used *Gwakhyangjeonggi-san* as a test liquid, too. 

The aim of this study is to establish a protocol of retention-enema experiments and evaluate the antihypertensive effect and the safety of retention enema using *Gwakhyangjeonggi-san*.

## 2. Materials and Methods

This study was carried out following National Institutes of Health Guide for the Care and Use of Laboratory Animals (NIH Publications no. 80-23, 1996, revised edition) and approved by Kyung Hee University Institutional Animal Care and Use Committee (KHUASP(SE)-12-039).

### 2.1. Plant Materials

A herbarium voucher sample (KHKH-2013-HK-118) that consists of *Gwakhyangjeonggi-san* was manufactured by Kyung Hee Herb Pharm and examined and deposited in the Korean Medicine Pharmaceutical Operation team, Department of Pharmacy, Kyung Hee University Korean Medicine Hospital (Seoul, Republic of Korea). And constituting components were analyzed in the Pharmaceutical Manufacturing Team, Department of Pharmacy, Kyung Hee University Korean Medicine Hospital (Seoul, Republic of Korea).

The herbs that compose *Gwakhyangjeonggi-san* are *Agastachis Herba* (above-ground parts) 6.0 g, *Perillae Herba* (leaves and twigs) 4.0 g, *Angelicae Dahuricae Radix* (root) 2.0 g, *Arecae Pericarpium* (peel) 2.0 g, *Hoelen* (sclerotium) 2.0 g, *Magnoliae Cortex* (bark) 2.0 g, *Atractylodis Rhizoma Alba* (rhizome) 2.0 g, *Citri Unshii Pericarpium* (peel) 2.0 g, *Pinelliae Tuber Cum Z*. *R*. *Crudus et Alumen * (tuber) 2.0 g, *Platycodi Radix* (root) 2.0 g, and *Glycyrrhizae Radix Preparata* (rhizome) 2.0 g. The total amount of the herbs is 28 g.

### 2.2. Extraction

We put all of the 11 chopped herbs into a felt pouch, then immersed the pouch into Pressure Oriental Herb Medicine Extractor (Handle-Type, KSNP B1130 - 240L, Kyungseo Machine, Incheon, Republic of Korea), and added 120 mL of water to submerge the herbs in the water. Using decocting method, it was concentrated for more than 2 hours until the volume reached 100 mL. The concentrated liquid was mixed with 0.9% saline and the proportion of the final test liquid used was 9 : 1 (concentrates : saline).

### 2.3. High Performance Liquid Chromatography (HPLC) Analysis

The HPLC system used consisted of Alliance 2690 Separation Module, a Waters 996 Photodiode Array Detector, and a Millenium 32 Chromatography Manager Version 3.2. Chromatographic separations were carried out on a Nucleosil C18 column (4.0 mm × 250 mm I.D. Waters Corporation, Milford, MA, USA) for glycyrrhizin, hesperidin, honokiol, and magnolol at ambient temperature.

Glycyrrhizin, honokiol, and magnolol were purchased from Wako pure chemicals industries, Ltd. (Osaka, Japan). Hesperidin was purchased from Sigma Co. (St. Louis, MO, USA). 

Acetonitrile (ACN) and water were used in the product of J. T. Baker (Phillipsburg, NJ, USA). Acetic acid and monopotassium phosphate were used in the product of Duksan pure chemicals Co., Ltd. (Ansan, Kyungki-do, Republic of Korea). 

The mobile phase on glycyrrhizin was water-ACN-acetic acid (620 : 380 : 5, v/v) with a flow rate of 1.2 mL/min. The mobile phase on hesperidin was 1/15 M monopotassium phosphate-ACN (815 : 185, v/v) with a flow rate of  1.0 mL/min. The mobile phase on honokiol and magnolol was ACN-water-acetic acid (500 : 500 : 10, v/v) with a flow rate of 1.5 mL/min. The mobile phase was filtered through a 0.45 *μ*m membrane filter (Millipore, Bedford, MA, USA) and was then degassed before use. 

GJS was used in the product of  Kyung Hee University Korean Medicine Hospital. Each sample was extracted with 50% ethanol for glycyrrhizin, methanol for hesperidin, and ethyl ether for magnolol and honokiol and filtered.

### 2.4. Animals

Male Sprague-Dawley rats (body weight = 300 ± 20 g) were used as normal and spontaneously hypertensive rats (SHRs). The normal rats were 7–9 weeks old, while the SHRs were 8 weeks old. Both were purchased from Samtaco Animal Corporation (Seoul, Republic of Korea). We adapted the experimental rats to the environment of laboratory for 7 days before the procedure. Less than 5 rats were raised in a plastic cage. We maintained the temperature (22°C ± 2°C) and the humidity (55%  ±  15 %) in the laboratory, giving the artificial sunlight for 12 hours every day. Distilled water and solid feed were given freely to rats for the experimental period. 

We divided 2 groups. The control group was defined as CON group (0.9% NaCl, 6 mL/300 g, *n* = 10), where retention enema with saline was performed. The other was GJS group (*Gwakhyangjeonggi-san*, 6 mL/300 g, *n* = 10), where retention enema with *Gwakhyangjeonggi-san* was performed. Ten rats were randomly allocated to each group in normal rats and SHR rats, respectively.

### 2.5. Final Protocol through a Preliminary Experiment

The training procedure was carried out 4 times a day and 30 minutes per time for 7 days to adapt the rats in the animal containment (Kent Scientific Corporation, USA) for measuring blood pressure. After the adaptive training, the normal rats and SHRs were relieved in a thermostat for 10~15 minutes. And we measured the body weight of the normal rats and SHRs, anesthetized the rats with isoflurane, fixed them on the operating table, and put them on anaesthetic masks in order. The syringe that contains test liquids, 0.9% of saline, was linked to an 8 Fr suction catheter and fastened up to a tube-connected insertion pump. With the anaesthetic masks worn, we reserved the test liquids for 10 minutes and then relived the rats again (Table 1).

### 2.6. Blood Pressure Measurement

**Figure 1:**
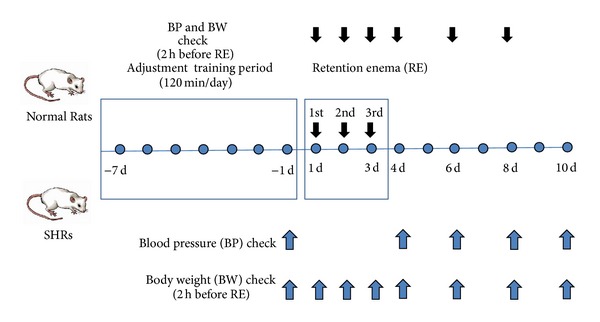
Experimental procedure.

We measured the rat blood pressure indirectly using a noninvasive blood pressure system (Kent Scientific Corporation, USA). By the tail-cuff method, the blood pressure was measured when not anesthetized after incubating at 36°C ± 0.2°C for 15 minutes [16]. Enema was performed 3 times in all of the rats. In normal rats, blood pressure was measured 2 hours before each enema and first, third, and fifth days after the last enema, whereas, in SHRs, on the day before the first enema and first, third, fifth, and seventh days after the last enema (Figure 1).

### 2.7. Body Weight Measurement

We measured body weight 3 times at 2 hours before each enema and at the same time on the first, third, and fifth days after the third enema in normal rats, whereas on the day before the first enema and on the first, third, fifth, and seventh days after the third enema in SHRs. 

### 2.8. Safety

#### 2.8.1. Experimental Group Allocation

We divided 3 groups, which are normal group (no intervention, *n* = 8), CON group (0.9% NaCl, 6 mL/300 g, *n* = 8), and GJS group (*Gwakhyangjeonggi-san*, 6 mL/300, *n* = 8), and randomly allocated 8 rats in each group. 

#### 2.8.2. Blood Sample Analysis

After 7-day adaptive training, we carried out a retention enema every day for 3 days. And we anesthetized the rats with isoflurane 2 hours after the third enema and collected 5 mL blood using a 26G disposable syringe by cardiac puncture method. The collected blood was put into SST bottle (BD vacutainer SST tube, BD Korea, USA) and analyzed. We requested aspartate aminotransferase (AST), alanine aminotransferase (ALT), blood urea nitrogen (BUN), and creatinine analysis to Eone Reference Laboratory (Incheon, Republic of Korea).

### 2.9. Statistical Analysis

We used the PASW Statistics 18.0 for Windows (Chicago, IL). Using generalized estimating equation, we tested group effect, time effect, and group × time effect between groups, as well as time effect within a group with the change of blood pressure and body weight, respectively. As of the safety, we applied Kruskal-Wallis test for a three-group analysis on variables with single assessment. And multiple comparisons using Mann-Whitney *U* test were performed with the differences of AST, ALT, BUN, and creatinine, respectively, between groups (employing two-tailed Bonferroni-corrected *P* value of <0.017 to minimize type I error). We calculated mean error and standard error and considered the errors significant when *P* < 0.05.

## 3. Result

### 3.1. Blood Pressure in Normal Rats

**Figure 2:**
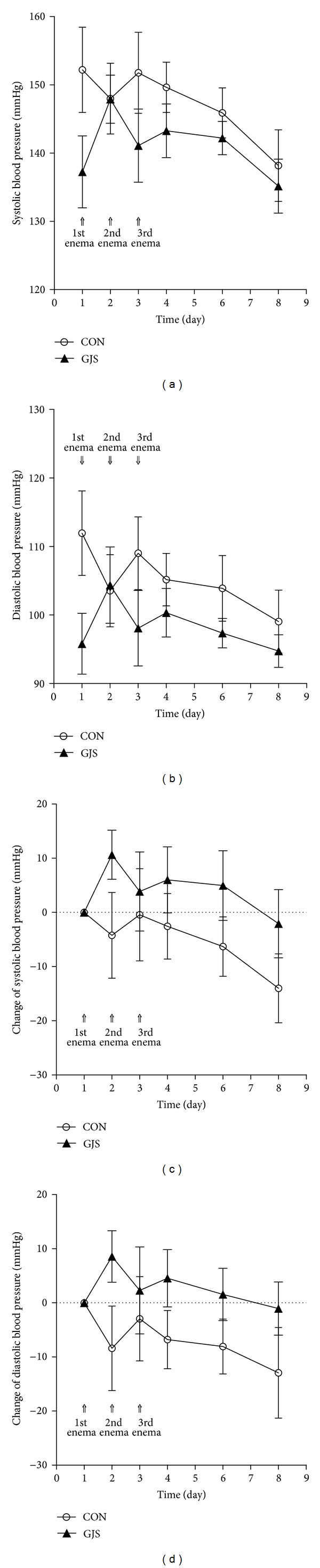
The blood pressure in the whole period. (a) Systolic blood pressure. (b) Diastolic blood pressure. (c) The change of systolic blood pressure. (d) The change of diastolic blood pressure.

Figure 1 shows the blood pressure measured in the whole period. The systolic blood pressure decreased in CON group, while it increased in the beginning and decreased gradually after all in GJS group (Figure 2(a)). The diastolic blood pressure showed the same tendency in both groups, respectively (Figure 2(b)). 

We calculated the difference of blood pressures daily, based on the baseline blood pressure day. The change of systolic blood pressure was negative in CON group but mostly positive on GJS group. And the absolute value of the systolic blood pressure increased in CON group, while it tended to decrease in GJS group during the whole period (Figure 2(c)). In the change of the diastolic blood pressure, the similar patterns appeared in both groups, respectively (Figure 2(d)).

In comparison of the systolic blood pressure between CON and GJS groups, there was no significant group × time effect (*P* = 0.374)  nor significant group effect (*P* = 0.179). But there was statistically significant time effect within CON group (*P* = 0.001) and GJS group (*P* < 0.001).

In comparison of the diastolic blood pressure between CON and GJS groups, there was no significant group × time effect (*P* = 0.180) nor significant group effect (*P* = 0.131). However, there was statistically significant time effect in GJS group (*P* = 0.001), but no significant time effect in CON group (*P* = 0.202).

### 3.2. Blood Pressure in SHRs

**Figure 3:**
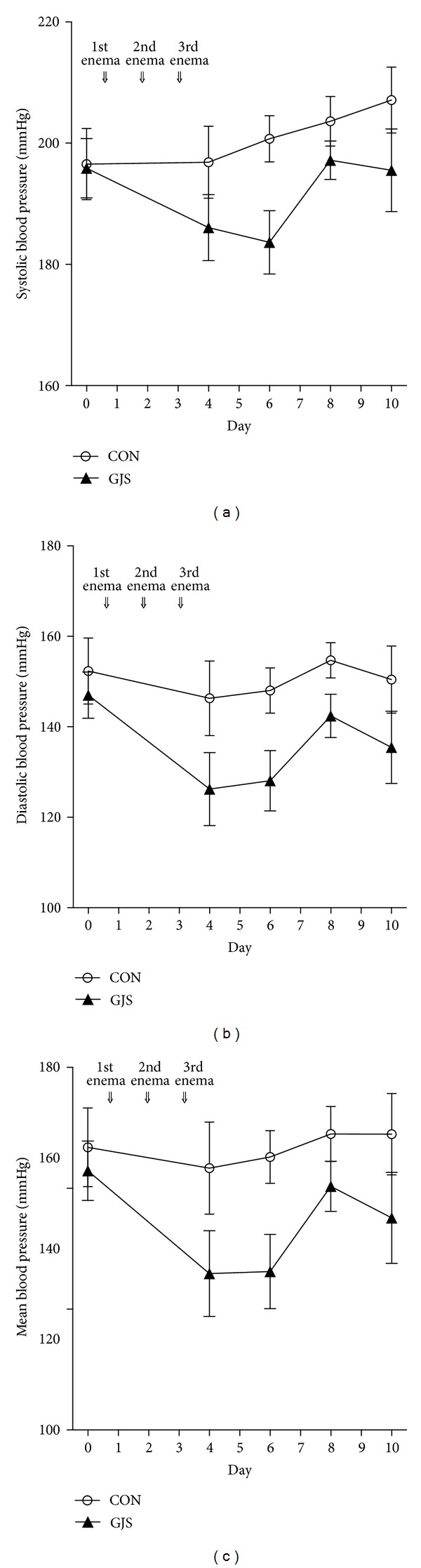
The blood pressure in the whole period. (a) Systolic blood pressure. (b) Diastolic blood pressure. (c) Mean blood pressure.

The systolic blood pressure increased during the whole period in CON group, while it decreased, except on the fifth day after 3 enemas, in GJS group (Figure 3(a)). The diastolic blood pressure in CON group increased overall, while it decreased, except on the third and fifth days after 3 enemas, in GJS group (Figure 3(b)). The mean blood pressure in CON group increased overall, while it fluctuated similarly with the pattern of the diastolic blood pressure in GJS group (Figure 3(c)).

The systolic blood pressure between CON and GJS groups did not show any significant difference in group × time effect (*P* = 0.396) but showed significant difference in the group effect (*P* = 0.012). And the time effect did not differ significantly in CON group (*P* = 0.471), while it differed significantly in GJS group (*P* < 0.001).

The diastolic blood pressure between CON and GJS groups did not show any significant difference in group × time effect (*P* = 0.572) but showed significant difference in group effect (*P* = 0.009). And the time effect did not differ significantly in CON group (*P* = 0.779), while it differed significantly in GJS group (*P* < 0.001).

The mean blood pressure between CON and GJS groups did not show any significant difference in group × time effect (*P* = 0.436) but showed significant difference in group effect (*P* = 0.012). And the time effect did not differ significantly in CON group (*P* = 0.956), while it differed significantly in GJS group (*P* < 0.001).

### 3.3. Body Weight in Normal Rats and SHRs

**Figure 4:**
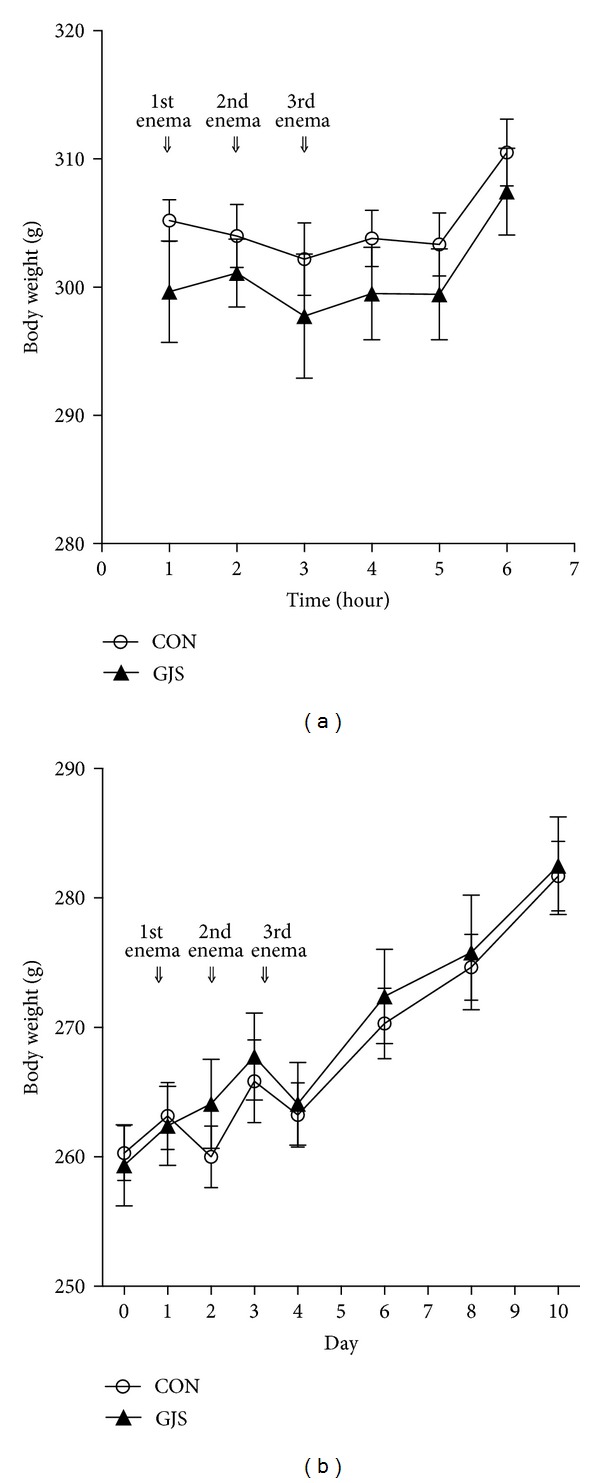
The body weight change in the whole period. (a) Normal rats. (b) SHRs.

The body weight in CON and GJS groups of normal rats decreased a little for 3 days when the enema was performed, maintained until the sixth day after the third enema, and increased after the eighth day (Figure 4(a)). No significant group × time effect existed (*P* = 0.715), and no group effect either (*P* = 0.363). However, the time effect appeared statistically significant in CON (*P* < 0.001) and GJS (*P* < 0.001) groups.

The body weight of SHRs increased overall during the whole period in both CON and GJS groups (Figure 4(b)). The group × time effect differed significantly (*P* = 0.001), so as the time effect within both groups (*P* < 0.001).

### 3.4. AST, ALT, BUN, and Creatinine

The measured AST was 112.25 ± 6.51 IU/L (normal group), 136.12 ± 20.09 IU/L (CON group), and 97.25 ± 3.94 IU/L (GJS group). The ALT was 33.25 ± 1.81 IU/L (normal group), 38.50 ± 2.16 IU/L (CON group), and 35.00 ± 1.31 IU/L (GJS group). The BUN was 14.55 ± 1.24 mg/dL (normal group), 16.83 ± 0.97 mg/dL (CON group), and 13.38 ± 0.94 mg/dL (GJS group). The creatinine was 0.27 ± 0.01 mg/dL (normal group), 0.27 ± 0.01 mg/dL (CON group), and 0.24 ± 0.01 mg/dL (GJS group). AST, ALT, BUN, and creatinine showed no significant difference between groups. 

### 3.5. HPLC Analysis

**Figure 5:**
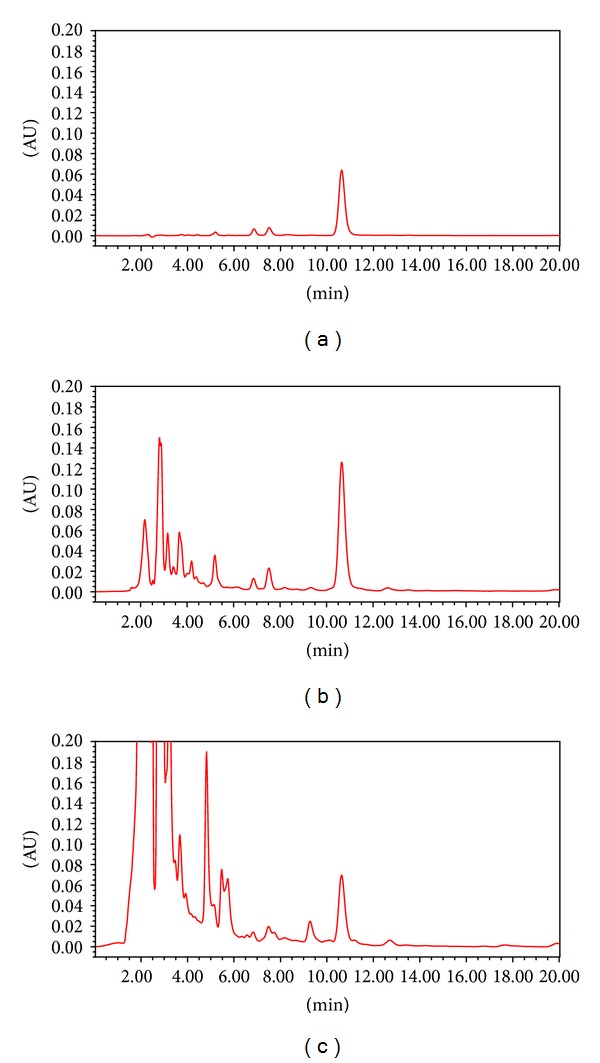
Chromatograms of (a) glycyrrhizin, (b) *Glycyrrhizae Radix*, and (c) GJS.

Figure 5 shows the typical chromatogram of glycyrrhizin (a), *Glycyrrhizae Radix* (b), and GJS (c). The peak time of glycyrrhizin was about 10.6 minutes and the content was 3.27% and 0.46% in *Glycyrrhizae Radix* and GJS, respectively. 

**Figure 6:**
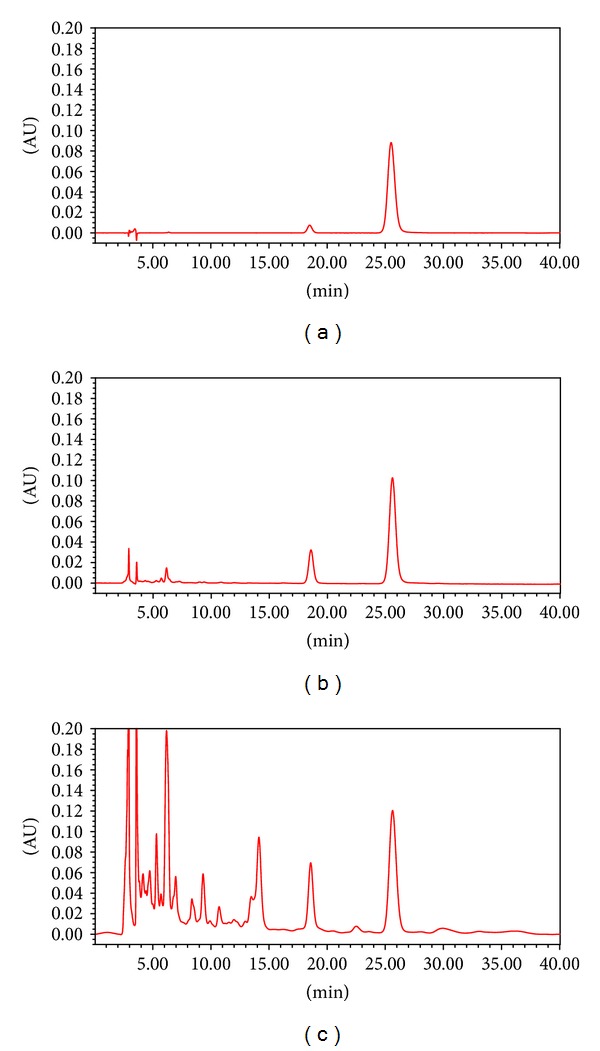
Chromatograms of (a) hesperidin, (b) *Citri pericarpium*, and (c) GJS.

Figure 6 shows the typical chromatogram of hesperidin (a), *Citri pericarpium* (b), and GJS (c). The peak time of hesperidin was about 25.5 minutes and the content was 4.7% and 0.6% in *Citri pericarpium* and GJS, respectively. 

**Figure 7:**
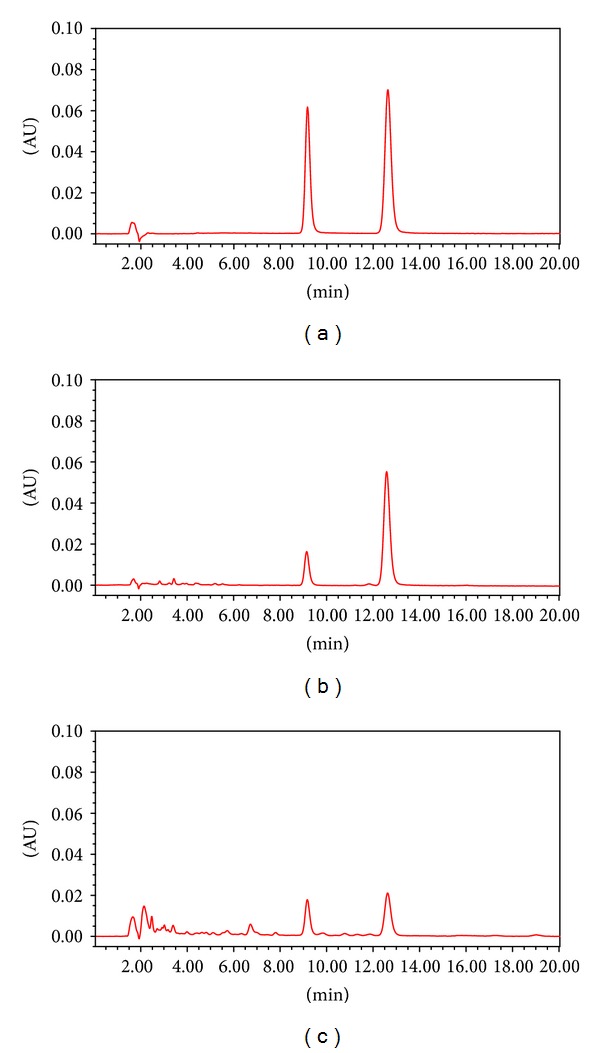
Chromatograms of (a) honokiol and magnolol, (b) *Magnoliae Cortex*, and (c) GJS.

Figure 7 shows the typical chromatogram of  honokiol and magnolol (a), *Magnoliae Cortex* (b), and GJS (c). The peak time of  honokiol was about 9.1 minutes and the content was 2.16% and 0.005% in *Magnoliae Cortex* and GJS, respectively. The peak time of magnolol was about 12.6 minutes and the content was 6.24% and 0.01% in *Magnoliae Cortex* and GJS, respectively. 

## 4. Discussion

We drew the protocol of retention enema through a pilot study and clinical experiences. The pilot study could make us confirm the efficacy and safety of the method. We used Sprague-Dawley rats because they are known to endure shock caused by enema and be easy to insert test liquids. As of the insertion tool and diameter, 6 Fr polyethylene catheter (male Wistar rats 250–300 g) [3] and 8 Fr pediatric foley catheter (S.D rat, 220–320 g) [5] have been applied. As of the insertion length, 6 cm [17] and 8 cm [7] have been used. About the retention time, the absorption concentration peaked around 10 minutes after the insertion in a study [18], and another researcher kept the test liquid for 10 minutes [6]. And the insertion velocity was 4 mL/min deprived from the running-fluid velocity, 1000 mL per 90 seconds, based on Kim's manual. The method applied in this study is very similar to the way used in the clinical practice.

A study reported that the test liquid made of *Gwakhyangjeonggi-san* is absorbed in the transverse colon during the retention [19]. However, the change of blood pressure did not differ between the CON groups and GJS groups in normal rats. This is because the normal rats do not have any disorders to be influenced by test liquids. On the other hand, the changes of systolic and diastolic blood pressure differed significantly with time in GJS group, and the changes of systolic blood pressure did in CON group too. This means that retention enema has hypotensive effect in normal rats. 

The systolic, diastolic, and mean blood pressure had no interactions between the group and time in CON and GJS groups of SHRs. However, the blood pressure decreased significantly with time only in GJS group, which indicates there is a meaningful antihypertensive effect when the *Gwakhyangjeonggi-san* retention enema is applied. This can be explained by a few reasons. First, *Gwakhyangjeonggi-san* is reported to have the vasodilator effect, which leads to the antihypertensive effect [15]. Second, pharmacologically, glycyrrhizin in *Glycyrrhizae Radix* can lower blood cholesterol level and blood pressure, hesperidin in *Citri pericarpium* has a hypotensive effect acting on smooth muscles in the blood vessel directly, and honokiol and magnolol in *Magnoliae Cortex* reduced the blood pressure of rats and cats by intravenous injection [20–22]. All of these components were analyzed by HLPC method in this study. Also it is known that angelic acid in *Angelicae Dahuricae Radix* stimulates the vasomotor center in medulla oblongata, the ether extract of *Hoelen* strengthens cardiac contraction, and *Platycodi Radix* lowers blood pressure for a short time [20–22], even though we could not measure 3 of these in our HPLC analysis. Third, the large intestine can absorb fats and oils components easily, which are contained a lot in herbs of *Gwakhyangjeonggi-san* [17]. Lastly, the test-liquid concentration absorbed in large intestine peaked in 10 minutes after the retention [18], which is applied to this study too. 

It is usually believed that the weight loss lower the blood pressure; however, the blood pressure decreased significantly in GJS group, even though the change of body weight increased in SHRs. This indicates the antihypertensive effect is from the test liquids, not from the weight loss. 

There was no significant difference in AST, ALT, BUN, and creatinine between groups. We found out that the retention enema by *Gwakhyangjeonggi-san*, which does not affect liver and kidney function, is a safe way. 

The importance of this study is that the protocol of retention enema has been established at first through the preliminary experiment and the clinical practice. Secondly, we have confirmed the safety of retention enema by *Gwakhyangjeonggi-san*, which means it does not affect the liver and kidney functions. Finally the antihypertensive effect has been revealed in *Gwakhyangjeonggi-san* retention enema. The effect maintained 7 days after the enema in SHRs.

The limitations are as follows. First, the baseline values of blood pressure were different between groups because of the different levels of adaptation by group in normal rats. We analyzed the change of blood pressures to correct the difference. Second, the observation time after the enema was too short. A long-term experiment is necessary to find out the continuous effect. Third, blood test to assess hepatic and renal enzymes, histological test to examine colonic mucosal changes, and additional test to identify intestinal bacteria are usually carried out in a safety study. However, only simple blood tests were performed with normal rats in this study. In the future, overall safety study will proceed including SHRs. Fourth, the HPLC results did not reveal every active component of every herb instituting *Gwakhyangjeonggi-san*. This could be because the lag of the HPLC machine or little mass of some herbs in *Gwakhyangjeonggi-san*. Fifth, considering the usual duration of drug activity, the maintenance of lowered blood pressure for 5 or 7 days could be due to the enema itself, not the enema with *Gwakhyangjeonggi-san*. Further studies are needed to investigate the precise mechanisms under the enema without any specific drugs. Lastly, the variation was too big because of the small sample. The sample size should be increased in the future study. 

This study suggests that the retention enema with *Gwakhyangjeonggi-san* is safe when performed in normal people, preventive of hypertension in prehypertension patients, and antihypertensive in hypertension patients. 

A long-term observation should be carried out in the future, based on which we can estimate the active duration of retention enema with *Gwakhyangjeonggi-san*. Secondly, the effect of the *Gwakhyangjeonggi-san* enema should be compared with that of the *Gwakhyangjeonggi-san *oral intake as a hypotensive effector. Finally, a randomized controlled trial should be performed to confirm the clinical effectiveness and safety.

## 5. Conclusions

The retention enema with *Gwakhyangjeonggi-san* has a hypotensive effect in normal rats within the regular range of blood pressure, but an antihypertensive effect in SHRs. The retention enema is safe and does not affect the liver and kidney functions in normal rats. 

## Figures and Tables

**Table 1:** The setting of drug retention enema.

	The kind, quality of the material and diameter of catheter	The insertion length of catheter	Sample volume	Retention time	Instillation speed
Setting	8 Fr suction catheter	8 cm	6 mL/300 g	10 min	4 mL/min

Procedure	(i) S.D rat (280–320 g)				
	(ii) Measuring BW				
	(iii) Anesthetizing by 2% isoflurane				
	(iv) Ventral position, angle (45°)				
	(v) Connecting syringe which is filled with sample or 0.9% saline to catheter and inserting it into the anal of S.D rat (8 fr suction catheter, 8 cm)				
	(vi) Plastering baseline in catheter				
	(vii) Instillation sample or 0.9% saline (6 mL/300, 4 mL/min)				
	(viii) Retaining sample or 0.9% saline during 10 min				
	(ix) Recovering S.D rat during 10 min (defecation)				
	(x) Measuring BW				
